# Control of the dynamics and homeostasis of the *Drosophila* Hedgehog receptor Patched by two C2-WW-HECT-E3 Ubiquitin ligases

**DOI:** 10.1098/rsob.150112

**Published:** 2015-10-07

**Authors:** Amira Brigui, Line Hofmann, Camilla Argüelles, Matthieu Sanial, Robert A. Holmgren, Anne Plessis

**Affiliations:** 1 Institut Jacques Monod, CNRS, UMR 7592, University Paris Diderot, Sorbonne Paris Cité, Paris 75205, France; 2 Department of Molecular Biosciences, Northwestern University, Evanston, IL 60208, USA

**Keywords:** *Drosophila*, signal transduction, intracellular protein trafficking, Hedgehog, Patched, C2-WW-HECT Ubiquitin ligase

## Abstract

The conserved Hedgehog (HH) signals control animal development, adult stem cell maintenance and oncogenesis. In *Drosophila*, the HH co-receptor Patched (PTC) controls both HH gradient formation and signalling. PTC is post-translationally downregulated by HH, which promotes its endocytosis and destabilization, but the mechanisms of PTC trafficking and its importance in the control of PTC remain to be understood. PTC interacts with E3 Ubiquitin (UB)-ligases of the C2-WW-HECT family; two of them—SMURF and NEDD4—are known to regulate its levels. We demonstrate that mutation of the PTC PY motif, which mediates binding of C2-WW-HECT family members, inhibits its internalization but not its autonomous and non-autonomous signalling activities. In addition, we show that the two related UB-C2-WW-HECT ligases NEDD4 and SU(DX) regulate PTC trafficking and finely tune its accumulation through partially redundant but distinct functions. While both NEDD4 and SU(DX) promote PTC endocytosis, only SU(DX) is able to induce its lysosomal targeting and degradation. In conclusion, PTC trafficking and homeostasis are tightly regulated by a family of UB-ligases.

## 1. Introduction

Hedgehog (HH) proteins play major roles in the development of many animals [1,2]. In *Drosophila*, HH controls the antero-posterior (A/P) patterning of the segments, and morphogenesis of numerous structures including the wing, eye and leg. This pathway is widely conserved in metazoans and its deregulation is involved in numerous pathologies in humans. Controlling HH signalling has therefore become a target for the treatment of cancers and cardiovascular diseases, for the repair of ischaemic tissues and more recently to improve cognitive function in Down syndrome patients [3–5].

HH reception relies on co-receptors, including the 12 transmembrane domain protein Patched (PTC). These proteins control, via the serpentine receptor Smoothened (SMO), a large cytoplasmic transduction complex that includes and regulates the transcription factor Cubitus interruptus (CI/GLI) [6]. In the absence of HH, PTC inhibits SMO, leading to the proteasome-dependent truncation of CI/GLI into a repressor form. HH binding to PTC and its co-receptors alleviates these negative effects on SMO, leading to SMO hyperphosphorylation, the inhibition of CI/GLI cleavage, the activation of full-length CI (CI^FL^) and the upregulation of HH target genes. The *ptc* gene is one of these targets, thus forming part of a negative feedback loop that controls the spreading of HH within responsive tissues [7,8].

Endocytosis of receptors is classically considered to be a means by which cells finely control signalling [9–11]. This process is used to tune the levels of the receptor accessible for the signal. By promoting the co-internalization and degradation of the bound ligand, it can also lead to signal termination or attenuation and shape the spread of morphogens. In some cases, receptor endocytosis was also reported to have positive effects on signalling [9–11]. The trafficking of transmembrane proteins often relies on their ubiquitination [12]. These post-translational modifications act as molecular signals that allow recognition by proteins of the endocytic or multi-vesicular body sorting machineries (see reviews in [13,14]). Therefore, the identification of the enzymes, Ubiquitin (UB) ligases and deubiquitinases that control receptor ubiquitination and the motifs that they recognize, along with understanding their regulation, is central to decipher the connection between receptor trafficking and activity.

HH signalling controls the dynamics of PTC and SMO, which shuttle from the plasma membrane and intracellular vesicles in flies, and in and out of the primary cilium in mammals. Indeed, experiments in *Drosophila* cells have shown that in the absence of HH, PTC is present both at the plasma membrane and in endosomes, while SMO is intracellular. By contrast, PTC bound to HH is endocytosed in a dynamin-dependent manner and targeted to the lysosome, while HH signalling leads to the stabilization of SMO at the cell surface due to a reduction of its endocytosis and/or an increase in its recycling back to the cell surface after internalization [7,15–17]. In mammals, HH controls the localization of SMO and PTC in and out of the primary cilium. Thus, in the absence of signal, PTC is localized at the cilium base where it prevents the accumulation of SMO into the cilium. HH reception leads to the movement of PTC out of the cilia and to the concentration of SMO into this structure.

Recent studies in flies have highlighted the importance of PTC and SMO post-translational modifications in the control of their trafficking. SMO accumulation at the plasma membrane requires its hyperphosphorylation by multiple kinases including the PKA and CKI. This is associated with a reduction of its ubiquitination, via the action of the deubiquitinase UBPY/UPS8 [18,19]. Moreover, several E3-UB-ligases of the C2-WW-HECT family proteins seem to bind and control PTC both in fly and in human cells [20]. The SMURF UB-ligase controls PTC ubiquitination and accumulation in *Drosophila* while its mammalian orthologues Smurf 1 and 2 regulate Ptc1 endocytic turnover and clearance from the primary cilium in response to HH [21,22].

We have previously identified, by yeast two-hybrid screens, several UB-ligases able to interact with the intracellular domains of *Drosophila* PTC [23]. Among these, NEDD4 (neural precursor cell expressed, developmentally downregulated 4), another C2-WW-HECT UB-ligase, interacts with the C-terminal tail of PTC. This interaction was confirmed by Lu *et al.* [24], who moreover showed that it requires a conserved L/PPXY (PY) motif known to promote interaction with E3 UB-ligase of the C2-WW-NEDD4 family [25,26]. The PY motif has also been implicated in PTC auto-regulating its own levels [27].

Here, using the *Drosophila* wing imaginal disc and fly-tissue-cultured cells as models, we further analysed the role of PTC trafficking and its control. Our study demonstrates that the PY motif of PTC is necessary for its internalization but is not absolutely required for its signalling functions. Moreover, we show that NEDD4 acts as a positive regulator of HH signalling by downregulating PTC levels and promoting its endocytosis. Finally, we uncover a role in HH signalling for a third fly C2-WW-HECT UB-ligase, Suppressor of deltex (SU(DX)), in the control of PTC endocytosis and its targeting to the lysosome. Altogether, our data support the conclusion that PTC trafficking is regulated by multiple UB-ligases, which finely tune its levels but are probably not essential for its signalling functions.

## 2. Results

### 2.1. The PY motif of Patched is required for its endocytosis

**Figure 1.**
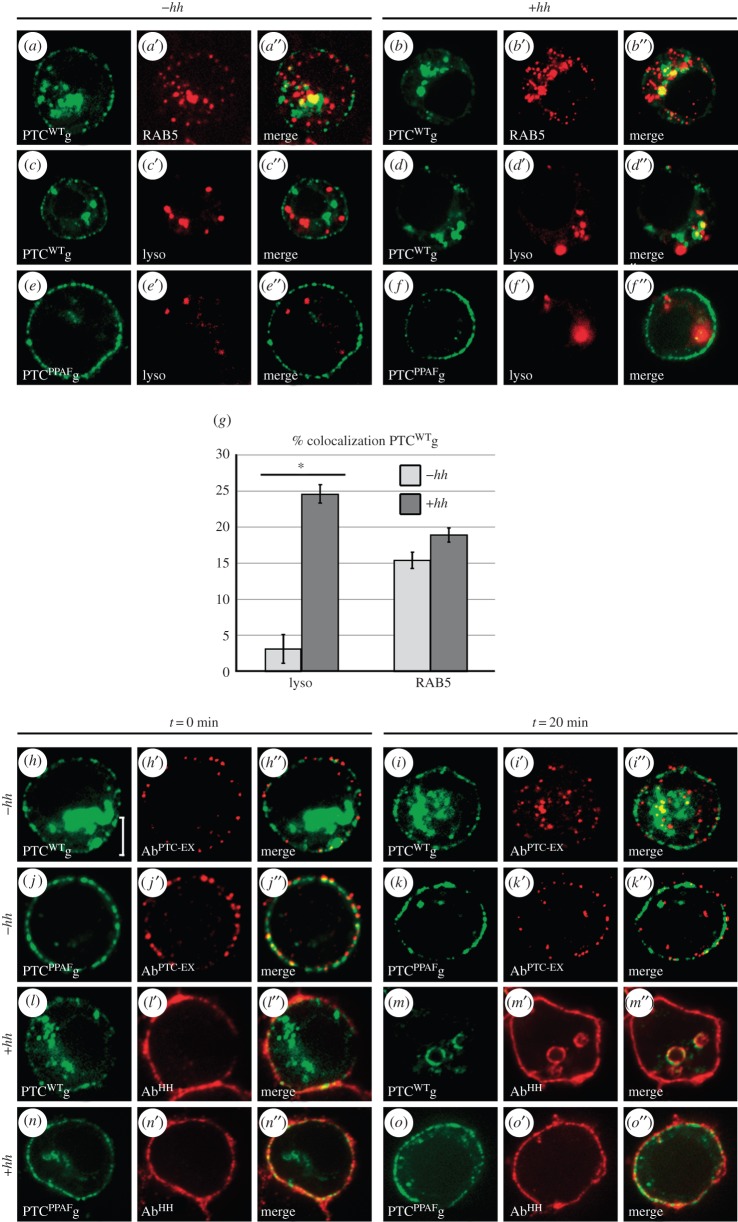
The PTC PY motif controls the accumulation and subcellular localization of PTC. (*a*–*f*) Confocal images showing the localization of PTC^WT^g or PTC^PPAF^g expressed in transfected S2 cells, in the presence (+) or absence (−) of HH. EE were immunolabelled for RAB5 (red, *a*′ and *b*′) and lysosomes were marked with a Lysotracker (lyso) (red, *c*′–*f*′). Note that the green fluorescence colocalizing with the Lysotracker may, at least in part, correspond to GFP remaining after PTC degradation in the lysosome. (*g*) Fraction of GFP+ (PTC^WT^g) vesicles colabelled for RAB5 or lyso. In all graphs, error bars correspond to standard deviation. HH promotes the targeting of PTC^WT^g to the lysosome but has no significant effect on PTC colocalization with Rab5. Data were from 250 images from 10 cells taken (with 25 non-overlapping images/cell). See also electronic supplementary material, table S1. See ‘Material and methods’ for further information on images treatment and quantification. Asterisk (*) indicates a statistically significant difference (*p* < 0.025). (*h*–*o″*) Internalization assay of PTC^WT^g or PTC^PPAF^g. Briefly, transfected S2 cells were incubated with a surface-bound antibody (Ab^PTC−EX^) directed against an extracellular epitope of PTC after endocytosis was transiently blocked by exposure to 4°C. Ab^PTC−EX^ was revealed either immediately after the cold block (*t* = 0) or 20 min after its release (by returning cells to 25°C) using fluorescent secondary antibodies (in red). See also electronic supplementary material, figure S1A,B. We noted that HH was still present at the plasma membrane even after PTC-WT endocytosis (m), possibly reflecting its anchoring to other transmembrane receptors such as Ihog and Boi. Ring-shaped internal structures were often seen in the presence of HH; some have vesicular structures and some seem to correspond to sections of tubes of the membrane after internalization. Their origin, nature and significance are unclear. In all cell images, scale bars are 5 µm.

The PY motif of PTC has been shown to control its stability and to interact with the C2-WW-HECT UB-ligases NEDD4 and SMURF [21,24]. We therefore assayed the role of the PY motif in the control of PTC subcellular localization. For that purpose, we mutated the PPAY sequence (aa 1205–08) into PPAF and we compared the subcellular localization of the wild-type (PTC^WT^) and mutant (PTC^PPAF^) forms of PTC in transfected S2 cells (figure 1*a*–*g*; electronic supplementary material, table S1). As expected, PTC^WT^ fused to GFP (PTC^WT^g) was at the plasma membrane and in early endosomes (EE): 15% of the PTC^WT^g was colabelled with RAB5 (figure 1*a*–*a*″,*g*) and only 3% with a lysosome dye that labels the acidic compartments (lysotracker, lyso; figure 1*c*–*c*″,*g*). HH induced PTC^WT^g targeting to the lysosome, as in this case almost 25% of PTC^WT^g was lyso+ (figure 1*d*–*d*″,*g*). Notably, the fraction of PTC^WT^g that was RAB5+ was not significantly affected (figure 1*b*–*b*″,*g*). In contrast, most of the PTC^PPAF^g was present at the plasma membrane even in the presence of HH (figure 1*e*–*e*″,*f*–*f*″). An endocytosis assay based on the uptake of an antibody directed against the PTC extracellular domain (Ab^PTC-EX^; electronic supplementary material, figure S1A) shows that this was due to reduced internalization of PTC^PPAF^g as opposed to increased recycling (figure 1*h*–*k*″; electronic supplementary material, figure S1B). A similar test with an anti-HH antibody also shows that HH was endocytosed in the presence of PTC^WT^g but not in the presence of PTC^PPAF^g (figure 1*l*–*o*″; electronic supplementary material, figure S1B).

Together these data show that PTC PY motif is required for its endocytosis in both absence and presence of HH.

### 2.2. Patched mutated for its PY motif over accumulates but is still active

**Figure 2.**
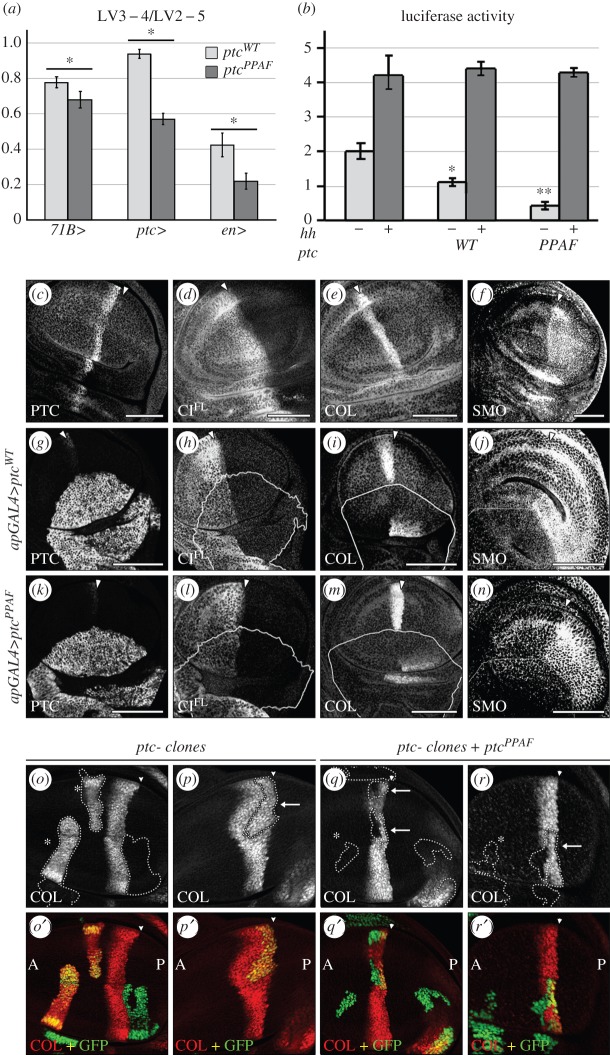
The PY motif controls PTC levels but is not essential for its function in HH signalling. (*a*) Relative LV3–4/LV2–5 spacing (normalized to MS1096 control wings) in wings of flies expressing *ptc^WT^* or *ptc^PPAF^* in the wing disc under the control of various drivers (as indicated). Expression of either construct led to a reduced LV3–4 spacing. The pattern of expression of the drivers and images of representative wings are shown in electronic supplementary material, figure S2A. (*b*) Expression of a *ptc-luc* reporter in transfected S2 cells is reduced by *ptc^WT^* or *ptc^PPAF^* expression. In the absence of HH, luciferase levels in the presence of *ptc^WT^* expression were statistically lower than in untransfected cells (*) but statistically higher than in cells transfected with *ptc^PPAF^* (**). These effects of *ptc^WT^* or *ptc^PPAF^* were suppressed in presence of HH. (*c*–*n*) Confocal images of third instar larvae wing discs expressing *ptc^WT^* or *ptc^PPAF^* with the *apGAL4* driver (active exclusively in the dorsal compartment). CI^FL^, COL or SMO detected by immunofluorescence (IF) were all downregulated, indicating that HH signalling is reduced. To ensure comparable expression levels, the *ptc^WT^* and *ptc^PPAF^* transgenes were inserted at the same locus. The accumulation of COL seen in the most ventral region, outside the wing pouch in (*i*) and (*m*), is reproducible but unexplained. The white line indicates the ventral limit of *apGAL4* expression (determined by PTC or GAL4 IF). Full arrowheads indicate the A/P boundary. In all figures, discs are oriented with A to the left and D to the bottom. (*o*–*r′*) Images of *ptc^16^/ptc^+^* disc with clones of *ptc^16^* mutant cells (marked by the GFP, green) expressing (*q*,*r*) or not (*o*,*p*) *ptc^PPAF^*. COL is labelled in red. Arrows: anterior clones near the boundary. Asterisk (*): clones further in the anterior compartment.

PTC endocytosis may play a role in the inhibition of SMO by PTC, the inhibition of PTC by HH and/or the range of HH diffusion and action. Previous findings suggested that the localization of PTC and SMO in the same endosomal compartment is necessary for the inhibition of SMO by PTC and that HH may antagonize the inhibitory effects of PTC by promoting its degradation [15,28]. However, previous analysis of PTC mutants with altered localization suggests that PTC at the plasma membrane could properly control HH signalling [7], but whether this mutant has increased recycling efficiency or a block in endocytosis is unknown. We took advantage of the fact that PTC endocytosis depends on its PY motif to assay whether it could influence its signalling properties. We compared the effects of overexpressing *ptc^PPAF^* or *ptc^WT^* in the *Drosophila* wing imaginal disc, the development of which is controlled by HH [29]. In this structure, HH produced by the cells of the posterior (P) compartment forms a gradient in the anterior (A) compartment, triggering the upregulation of target genes, such as *collier* (*col*), *decapentaplegic* (*dpp*) and *ptc* itself. Expression of either *ptc^WT^* or *ptc^PPAF^* throughout the wing imaginal disc (*71BGAL4* driver) or in HH-responsive cells along the A/P boundary (*ptcGAL4* driver) reduced spacing between the longitudinal veins (LV) 3 and 4, a mark of lower levels of HH signalling (figure 2*a*; electronic supplementary material, figure S2A). Accordingly, overexpression of either transgene in the dorsal (D) compartment of the wing imaginal disc (*apGAL4* driver) reduced the accumulation of CI^FL^ (figure 2*d*,*h*,*l*) as well as SMO (figure 2*f*,*j*,*n*), which are both normally stabilized by HH (figure 2*d*,*f*). It also reduced the expression of *col*, a gene whose expression is upregulated by high levels of HH signal (figure 2*e*,*i*,*m*). These negative effects of PTC^PPAF^ on HH signalling did not depend on the presence of the endogenous PTC protein. Indeed, as shown in figure 2*o–r*′, PTC^PPAF^ expression prevented the ectopic *col* expression that is normally induced in anterior clones mutant for *ptc* and downregulated the endogeneous expression of *col* at the A/P boundary. These results extend Casali's previous report, which showed that a PTC mutant with its PPAY motif replaced by AAAA did not have altered expression of *dpp*, a gene activated by low levels of HH signalling [27]. Together these data show that PTC^PPAF^ has kept the ability to reduce HH signalling.

Within the *ptc^−^* clones expressing PTC^PPAF^, *col* expression remained in the cells nearest to the A/P border where the highest levels of HH are present (arrows in figure 2*q*–*r*). Additionally, in S2 cells, the negative effects of either PTC^PPAF^ or PTC^WT^ on a HH-responsive reporter (*ptc-luc* [30]) were both suppressed by HH (figure 2*b*). Thus, PTC^PPAF^ has kept the ability to be regulated by HH, indicating that HH can antagonize a form of PTC that cannot be endocytosed.

Another aspect of PTC function is to shape the HH gradient by promoting its co-internalization [8]. Thus, ectopic expression of *col* occurs in cells that anteriorly abut *ptc^−^* clones along the A/P compartment boundary (figure 2*p*) due to increased HH spreading across the clone [8]. This non-autonomous effect of *ptc^−^* loss of function is however suppressed when PTC^PPAF^ is expressed within the clone (figure 2*q*,*r*), indicating that it has kept the ability to restrain HH spreading. This was confirmed by the fact that PTC^PPAF^ overexpression in the P compartment of the wing imaginal disc (using *enGAL4* driver) reduced HH signalling (figure 2*a*; electronic supplementary material, figure S2A).

All these data indicate that PTC can both restrain HH diffusion and be inhibited by HH independently of its PY motif.

Despite the fact that *ptc^WT^* and *ptc^PPAF^* were expressed under the same conditions from the same locus, we noted that *ptc^PPAF^* had a consistently stronger effect than *ptc^WT^* in many of the above *in vivo* experiments (figure 2*a*; electronic supplementary material, figure S2A). This difference in effect was confirmed in S2 cells, using the HH-inducible luciferase reporter (figure 2*b*). Notably, the levels of PTC^PPAF^ fused to a HA tag (PTC^PPAF^-HA) were five times higher than those of PTC^WT^-HA (electronic supplementary material, figure S2B). This is in agreement with what was observed by Lu *et al.* [24] for another mutant of the PY motif (see also electronic supplementary material, figure S2C, for a further comparison) and may be sufficient to account for the stronger effects of PTC^PPAF^.

In summary, PTC PY motif is not required for PTC signalling activity, but is important for its homeostasis by controlling its total levels and its levels at the cell surface, where it can receive HH.

### 2.3. The E3 Ubiquitin-ligase NEDD4 controls Patched internalization and accumulation levels

**Figure 3.**
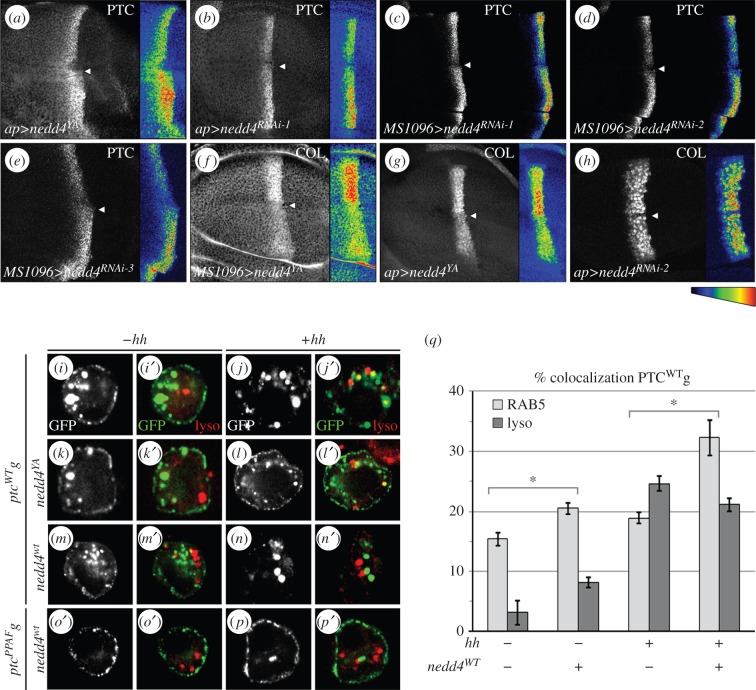
NEDD4 downregulates PTC and promotes its internalization. (*a*–*h*) Confocal images of wing disc immunolabelled for (*a*–*e*) PTC or (*f*–*h*) COL and expressing a dominant-negative form of *nedd4* (*nedd4^YA^*) (*a*) or three different *nedd4^RNAi^* (1, 2, 3; see ‘Material and methods’) with *MS1096GAL4* (which is more expressed in the dorsal compartment than in the ventral one), or *apGAL4* (exclusively expressed in the dorsal compartment). Staining intensities in pseudocolour are shown on the right. In all cases, downregulation of NEDD4 activity led to an upregulation of PTC accumulation and a reduction in *col* expression. The effect of *nedd4^RNAi2^* on *col* was visible in the cells near the D/V boundary, in a region known to be sensitive to small changes in HH signalling. Arrowhead: D/V boundary. (*i*–*p*′) Confocal images of S2 cells expressing *ptc^WT^g* or *ptc^PPAF^*g with *nedd4^YA^* (*k*–*l*′) or *nedd4^WT^* (*m*–*p*′) and colabelled with the Lysotracker (lyso). (*i*–*j*′) are control cells with *ptcWTg* alone*.* (*q*) Quantification of the effect of *nedd4^WT^* on the fraction of PTC^WT^ that colabelled with RAB5 (light grey) or the Lysotracker (lyso, dark grey).

NEDD4 is widely expressed in *Drosophila*, including the wing imaginal disc, and in S2 cells [31–33]. In cotransfected S2 cells, NEDD4 is present both at the cell membrane and in large vesicular structures, many of them colabelled with PTC^WT^g (electronic supplementary material, figure S3A). We decreased NEDD4 activity throughout the wing disc either by expression of a catalytically inactive form (*nedd4^YA^*) known to act in a dominant-negative fashion or by RNA interference. Both methods have previously been shown to downregulate endogenous NEDD4 activity [31–33]. Moreover, we also used two other independent RNAi constructs directed against different regions of *nedd4* mRNA. Downregulation of NEDD4 activity by expression of any of the four transgenes led to the overaccumulation of the endogenous PTC protein (figure 3*a*–*e*). This was probably due to a post-translational regulation of PTC levels, rather than an upregulation of its transcription resulting from increased HH signalling. Indeed, the LV3–4 spacing was reduced (electronic supplementary material, figure S3B,C) and the expression of *col* was attenuated (figure 3*f*–*h*), both of which indicate that HH signalling was reduced. This conclusion was further supported by the fact that expression of *nedd4^YA^* also led to increased accumulation of PTC in S2 cells in which *ptc* transcription cannot be upregulated, due to the lack of CI expression (electronic supplementary material, figure S3E).

We next tested whether NEDD4 could regulate PTC trafficking using S2 cells. Expression of *nedd4*^YA^ led to the accumulation of PTC^WT^g at the plasma membrane, in both the presence and absence of HH (figure 3*k*–*l*′, compare to *i*–*j*′). Internalization of the Ab^PTC−EX^–PTC^WT^g complex was also inhibited in these cells, confirming that PTC endocytosis was blocked (electronic supplementary material, figures S1B and S4c–d″). Conversely, cells overexpressing *nedd4*^WT^ showed a reduced accumulation of PTC^WT^g at the cell surface (figure 3*m*–*n*′) as a result of increased PTC internalization (electronic supplementary material, figures S1B and S4a–b″). This effect was dependent on the PY motif of PTC, even in the presence of HH (figure 3*o*–*p*′; electronic supplementary material, figures S1B and S4i–j″). Moreover, *nedd4*^WT^ overexpression increased PTC^WT^g localization to the EE (20% of PTC^WT^g vesicles were RAB5+) but had a weak effect on the targeting to the lysosome (8% of PTC^WT^g vesicles were lyso+; figure 3*q*; electronic supplementary material, table S1).

In conclusion, our data indicate that NEDD4 is a positive regulator of HH signalling that directly controls the level of PTC accumulation at the plasma membrane by promoting its endocytosis.

### 2.4. The E3 Ubiquitin-ligase SU(DX) regulates Hedgehog signalling and controls Patched sorting to the lysosome

**Figure 4.**
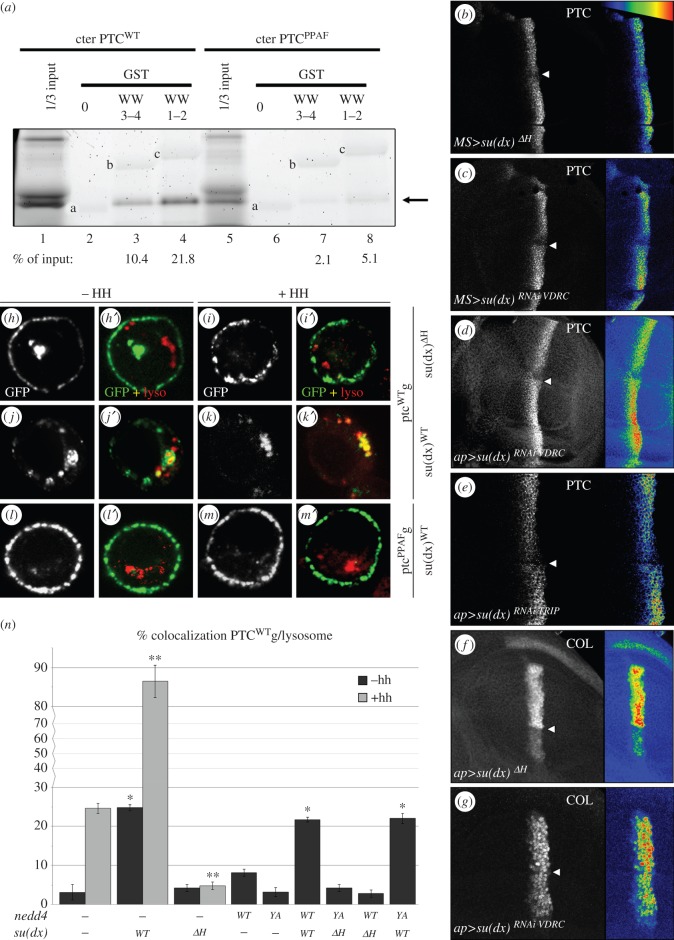
SU(DX) downregulates PTC and controls both its endocytosis and sorting to the lysosome. (*a*) A GST pull-down assay was performed between the *in vitro* translated, fluorescently labelled C-terminal (C-ter) domain of PTC^WT^ (lanes 1–4) or PTC^PPAF^ (lanes 5–8) and GST alone (lanes 2 and 6), or GST fused to the WW domains (WW1–2 or 3–4) of SU(DX) (lanes 3–4 and 7–8). One-third of the amount used in the pull-down assays was loaded as inputs (lanes 1 and 6). The fraction (%) of PTC C-ter pulled-down is indicated under each corresponding lane. It shows that PTC^WT^ but not PTC^PPAF^ interacts with SU(DX). Arrow: PTC-ter. The bands a, b and c correspond to the GST, GST-WW3–4 and GST-WW1–2, respectively, which emitted low fluorescence under our experimental conditions. (*b*–*g*) Confocal images of wing discs immunolabelled for (*b*–*e*) PTC or (*f*–*g*) COL and expressing (*b*,*f*) a dominant-negative form of *su(dx)* (*su(dx)^ΔH^*) or (*c*–*e*,*g*) two different *su(dx)^RNAi^* transgenes (VDRC and TRIP; see ‘Material and methods’) through the *MS1096GAL4* or *apGAL4* drivers. In all cases, downregulation of SU(DX) led to an upregulation of PTC accumulation and a reduction in COL expression. (*h*–*m*′) Confocal images of S2 cells expressing (*h*–*k*′) *ptc*^WT^g or (*l*–*m*′) *ptc*^PPAF^g (green), along with *su(dx)^ΔH^* or *su(dx)^WT^* (as indicated) and colabelled with the Lysotracker (red). (*n*) Quantification of the fraction of PTC colabelled with the Lysotracker dye in S2 cells transfected with *ptc^WT^g* alone or in various combinations with *su(dx)^WT^*, *su(dx)^ΔH^*, *nedd4^WT^* or *nedd4^YA^* as indicated.

The absence of significant effects of NEDD4 on the lysosomal targeting of PTC suggests that other UB-ligases could control this step. The endocytosis and post-endocytic sorting of transmembrane proteins, such as Notch, have been shown to be regulated, respectively, by NEDD4 and Itch/AIP4/Suppressor of deltex (SU(DX)), a NEDD4-related UB-ligase [32–34], and this could also be the case for PTC. *su(dx)* is expressed in all cells of the wing imaginal disc and in S2 cells [32], where it colocalizes with PTC in vesicular structures (electronic supplementary material, figure S3A). Moreover, the C-terminal domain of PTC interacts via its PY motif with the WW domains of SU(DX) *in vitro* (figure 4*a*). We therefore examined whether SU(DX) could also regulate PTC and HH signalling. Reducing SU(DX) activity by expressing two different RNAi constructs—directed against two different regions of *su(dx)*, (*su(dx)^RNAi^^VDRC^* and *su(dx)^RNAi^^TRIP^*)—or a transgene encoding a dominant-negative HECT domain deletion (*su(dx)*^ΔH^) [32,35] led to an increase in PTC protein accumulation at the A/P boundary (figure 4*b*–*e*). This effect was associated with a decrease in *col* expression and a reduction in LV3–4 spacing (figure 4*f*–*g*; electronic supplementary material, figure S3B,D), both indicating a reduction in HH signalling.

Next, we analysed the effects of *su(dx)* overexpression on PTC trafficking using S2 cells. PTC^WT^g accumulation and endocytosis were blocked by *su(dx)*^ΔH^ expression, with or without HH (figure 4*h*–*i*′; electronic supplementary material, figure, S1B and S4 g–h″). By contrast, *su(dx)*^WT^ overexpression led to a reduced accumulation of PTC^WT^g at the plasma membrane due to PY-dependent increase in PTC internalization (figure 4*j*–*m*′; electronic supplementary material, figures, S1B and S4e–f′,k–l″). Moreover, *su(dx))*^WT^ expression strongly enhanced PTC targeting to the lysosome as the fraction of GFP+ vesicles that were also lyso^+^ increased to almost 25% in absence of HH and reached 86% with HH (figure 4*n*; electronic supplementary material, table S1).

Finally, to better understand the respective role of NEDD4 and SU(DX), we expressed combinations of the wild-type and mutant forms of these two UB-ligases (figure 4*n*; electronic supplementary material, figures S4 m–t″ and S1B, and table S1). In S2 cells, coexpressing either *nedd4^WT^* with *su(dx)^ΔH^* or *nedd4^YA^* with *su(dx)^WT^*, PTC^WT^g was still internalized, suggesting possible redundant functions for these two proteins in PTC endocytosis in these cell. Co-expression of *nedd4^YA^* and *su(dx)^WT^*, but not of *nedd4^WT^* and *su(dx)^ΔH^*, promoted the targeting of PTC^WT^g to the lysosome at a level similar to that induced by the overexpression of *su(dx)^W^*^T^ alone. This further indicates a specific role for SU(DX) in sorting PTC to the lysosome.

## 3. Discussion

### 3.1. Multiple Ubiquitin ligases control Patched trafficking

During its journey from the plasma membrane to the lysosome or to a recycling pathway, an integral membrane receptor can undergo several rounds of ubiquitination and deubiquitination, each controlling a particular trafficking step [36,37]. In yeast, these ubiquitination steps are catalysed by the same and specific C2-WW-HECT ligase, Rsp5p [13]. In multicellular eukaryotes, these events may involve multiple UB ligases of the HECT and RING (Really Interesting New Gene) families [38,39]. The accumulation of the HH receptor PTC seems to be regulated by the complex action of the three C2-WW-HECT UB-ligases present in fly. We show that NEDD4 and SU(DX) finely regulate PTC homeostasis both in S2 cultured cells and *in vivo*, in the wing imaginal disc. We provide evidence that in cultured cells, these two related UB-ligases differentially control PTC internalization and targeting to the lysosome. While NEDD4 and SU(DX) control the internalization of PTC both in the presence and in the absence of HH ligand, only SU(DX) acts on PTC *en route* to the lysosome, this latter effect being highly enhanced in presence of HH signal. The third UB-ligase of the same family, SMURF, was also reported to induce PTC degradation in a manner that depends on SMO activation, but how it impacts PTC trafficking remains to be determined [21].

The mechanisms underlying the distinct actions of these related UB-ligases remain to be understood. It may be related to differences in the UB chain formed, as in mammals, Itch/AIP4 has been reported to catalyse Notch polyubiquitination through unusual K29-linked chains [40], indicating a link between this type of chain and lysosomal degradation [41]. Alternatively, these differences could be the consequence of the different subcellular localization of these ligases and/or reflect changes in their interaction with PTC at different stages of the endocytic pathway via the recruitment of specific adaptor proteins or changes in post-translational modification [42–46].

### 3.2. Patched endocytosis controls its homeostasis

A key issue regarding PTC is the role of trafficking in the control of its activity. To answer such a question requires tools that specifically target PTC trafficking as opposed to blocking the endocytic machinery in general, which would be likely to have pleiotropic effects. Using a PTC mutant specifically impaired for its internalization due to the mutation of its PY motif, we provide further proof that PTC trafficking is not necessary for its intrinsic signalling properties. This implies that PTC colocalization with SMO in endosomes is not required for PTC to inhibit SMO and that HH can inhibit PTC independently of promoting its degradation. This differs with observations made for other receptors such as Frizzled, Notch or EGFR, for which intact trafficking is important in productive signalling [9,10]. Nonetheless, PTC trafficking plays a major role in controlling its levels (in both the presence and absence of HH), which is important for the finely tuned control of both HH pathway activity and HH gradient formation. Noteworthy, three other UB-ligases unrelated to NEDD4, SU(DX) and SMURF were also found to interact with PTC through a high-throughput protein interaction screen approach [23]. The multiplicity of the UB-ligases potentially involved in the control of PTC homeostasis could reflect redundancy and/or differential use in different biological contexts, and highlight the crucial importance of controlling of PTC levels for proper development.

### 3.3. Broader implications

Strikingly, the hPTC proteins contain two PY motifs that can bind numerous C2-WW-HECT UB-ligases homologous to NEDD4 and SU(DX) [20,22]. It was recently reported that the mutation of these PY sites prevents the HH-induced removal of hPTC from the primary cilia in response to HH [20]. This shows that, despite the apparent divergence of the pathway and the requirement for the primary cilium in mammals, the same processes are used to control the internalization of PTC from the plasma membrane in flies, and its removal from the primary cilium shaft in mammals. Moreover, similar to our observations in flies, these mutations do not prevent PTC from negatively regulating SMO nor responding to HH, confirming that PTC internalization is not crucial for HH signalling. PTC is related to transmembrane transporters and has been proposed to control SMO activity via lipids. The fact that it can act independently of its own trafficking constrains how this might take place and suggests that HH might regulate PTC transport activity by promoting structural changes and/or a so-far-unknown post-translational modification.

It is noteworthy that the Nieman-Pick1 (NPC1) protein, which is involved in NPC disease (a fatal lysosomal storage disorder) and is also related to PTC, contains an intracellular PY motif and has highly dynamic trafficking. Thus, the present characterization of specific proteins and the sequence involved in the regulation of PTC trafficking is very likely to be applicable to the other members of the PTC/NPC family and should have important implications for their regulation.

## 4. Material and methods

### 4.1. Fly strains and genetics

Flies were raised at 25°C. *pUASt-AttB-ptc^WT^* and *pUASt-AttB-ptc^PPAF^* transgenes were inserted by the фC31 method [47] in the *Drosophila* line 9725 [48], which contains an *attP* site at position 75A10. Injections were carried out by BestGene Inc. Transgenes of interest were expressed using the UAS-GAL4 system [49]. Fly strains were described previously, as follows: *UASt-nedd4^WT^* (chr. II) [33], *UASt-nedd4^YA^* (chr. II) [33], *UASt*-*nedd4^RNAi^*^1^ (chr. II from [31]), *UASt*-*nedd4^RNAi^*^2^ (chr. III, from Bloomington, stock 31687) *UASt*-*nedd4^RNAi^*^3^ (chr. III, from Bloomington, stock 34741), *UASt-sudx^WT^* (chr. II) [32], *UASt-sudx^ΔH^* (chr. II) [32], *UASt*-*su(dx)^RNAi^* ^VDRC^ (chr. II, from the VDRC, 102798 insertion), *UASt*-*su(dx)^RNAi^* ^TRIP^ (chr III, from Bloomington, stock 36836), *MS1096GAL4* (chr. X) [50], *71BGAL4* (chr. III) [49], *apGAL4* (chr. II) [51], *enGAL4* (chr II, gift from P. Thérond)*.* MARCM clones: *FRT42D ptc^16^/CyO*; *HMC/TM6b* or *FRT42D ptc^16^/CyO*; *UAS ptc^PPAF^* females were crossed to *y*, *w*, *hsFLP*, *tub-GAL4 UAS-gfp/Y*; *tub-GAL80^ts^ FRT42D/CyO* males; larvae were incubated 1 h at 37°C for three consecutive days to induce mosaic clones.

### 4.2. Quantification of PTCg colabelling with vesicular markers

The proportion of green pixels (PTCg) superimposing with pixels labelled in red (RAB5 or Lysotracker) was determined on untreated images. Automated quantification was performed with ImageJ software; vesicle segmentation was carried out using grey level thresholds on wavelet-transformed images. To calculate the percentage of colocalization occurring by chance, PTCg images were rotated by 90° respective to their matching vesicular marker images and re-examined for colocalization with markers. We found that less than 5% of PTCg randomly colocalized with any given marker. For each vesicular marker, we measured 10 cells (with 25 images per stack) selected randomly from three independent transfections. Note that a similar PTCg construct was previously reported to rescue a *ptc* null mutation [7].

### 4.3. Endocytosis assay

Forty-eight hours after transfection, S2 cells were placed on ice for 10 min, before being incubated for 20 min with anti-PTC antibody (Apa1 [50], 1/500). After washing the unbound antibody, half of the cells were plated on Concanavalin A (Sigma) coated glass coverslips for 5 min before fixation for 30 min with 4% paraformaldehyde. The other half was further incubated for 20 min at 25°C before plating on Concanavalin A and fixation. The two samples were then permeabilized three times for 5 min in PBS + 0.1%Tween (PBST) and incubated for 1 h with the secondary antibody. This was followed by incubation for 5 min with DAPI and three washes in PBST, before mounting in Citifluor.

## Supplementary Material

Figure S1: Control of PTC trafficking.

## Supplementary Material

Figure S2: Impact of mutating the PY motif of PTC on its activity and accumulation.

## Supplementary Material

Figure S3: NEDD4 and SU(DX) colocalize with PTC and their downregulation affects wing morphogenesis.

## Supplementary Material

(A) Images of S2 cells showing the colocalization of PTCWTg (green) and NEDD4 (immunolabeled in red) at the cell surface and in vesicles (a-b”) or of endogenous PTC (green) and SU(DX) (red) in vesicles (c-d”). Without HH (a-a”, c-c”) or with HH (b-b”, d-d”). (B-D) Wings from transgenic flies overexpressing nedd4YA (a), nedd4RNAi1 (b), nedd4wT (c), su(dx)ΔH (d), su(dx)RNAiVDRC (e) and su(dx)WT (f) driven by MS1096GAL4. The reduced LV3-4 spacing (measured in C and D) seen when nedd4 or su(dx) expression or activity are reduced is typical of inhibition of this pathway. Oppositely, nedd4 or su(dx) overexpression leads to an increase of the LV3/LV4 spacing (measured in C and D) as expected from an activation of HH signaling. The other effects observed can be attributed to the previously reported effects of these two HECT ligases on the Notch pathway. Note that downregulation of nedd4 or of su(dx) resulted in different phenotypes and that the effects observed for expression of the dominant-negative form of each ligase were similar to those observed following their knockdown using RNAi (compare a/b to d/e). These observations supports the specificity of the RNAi and dominant negative tools used here. (E) Immunodetection of PTC-HA in extracts of S2 cells expressing ptcWT without and with nedd4YA.

## Supplementary Material

Table S1: Quantitication of the colocalization of PTCWTg with RAB5 or Lyso.

## Supplementary Material

Table S1 legend.docx

## Supplementary Material

Sup mat and meth 8 sept 2015.docx
