# Supplementary figures and images for: Control of the dynamics and homeostasis of the Drosophila Hedgehog receptor Patched by two C2-WW-HECT-E3 Ubiquitin ligases
Source: Open Biol. 2015 Oct 7;5(10):150112. doi: 10.1098/rsob.150112 (PMC4632511; doi:10.1098/rsob.150112)

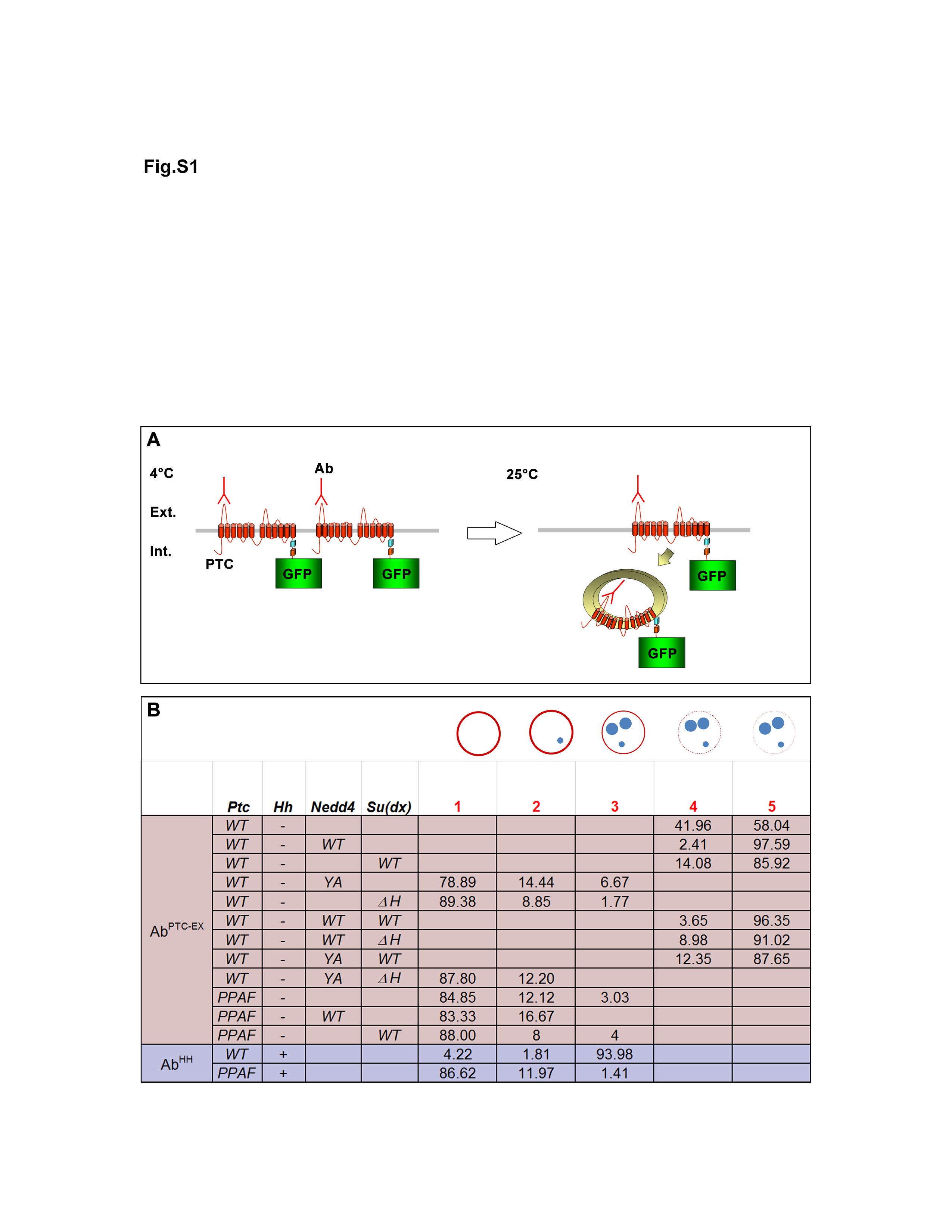

Supplement: Figure S1: Control of PTC trafficking. [file rsob150112supp1.tif]

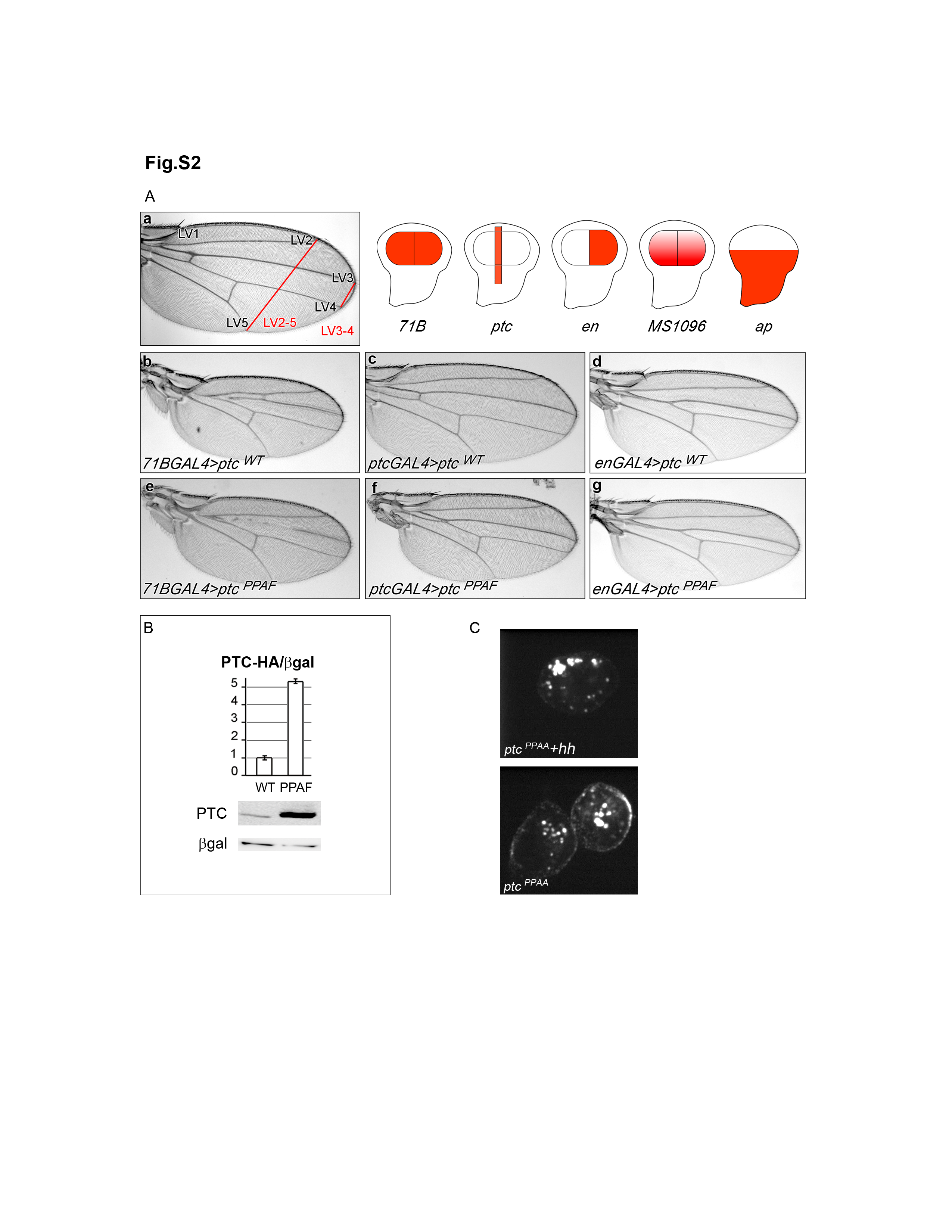

Supplement: Figure S2: Impact of mutating the PY motif of PTC on its activity and accumulation. [file rsob150112supp2.tif]

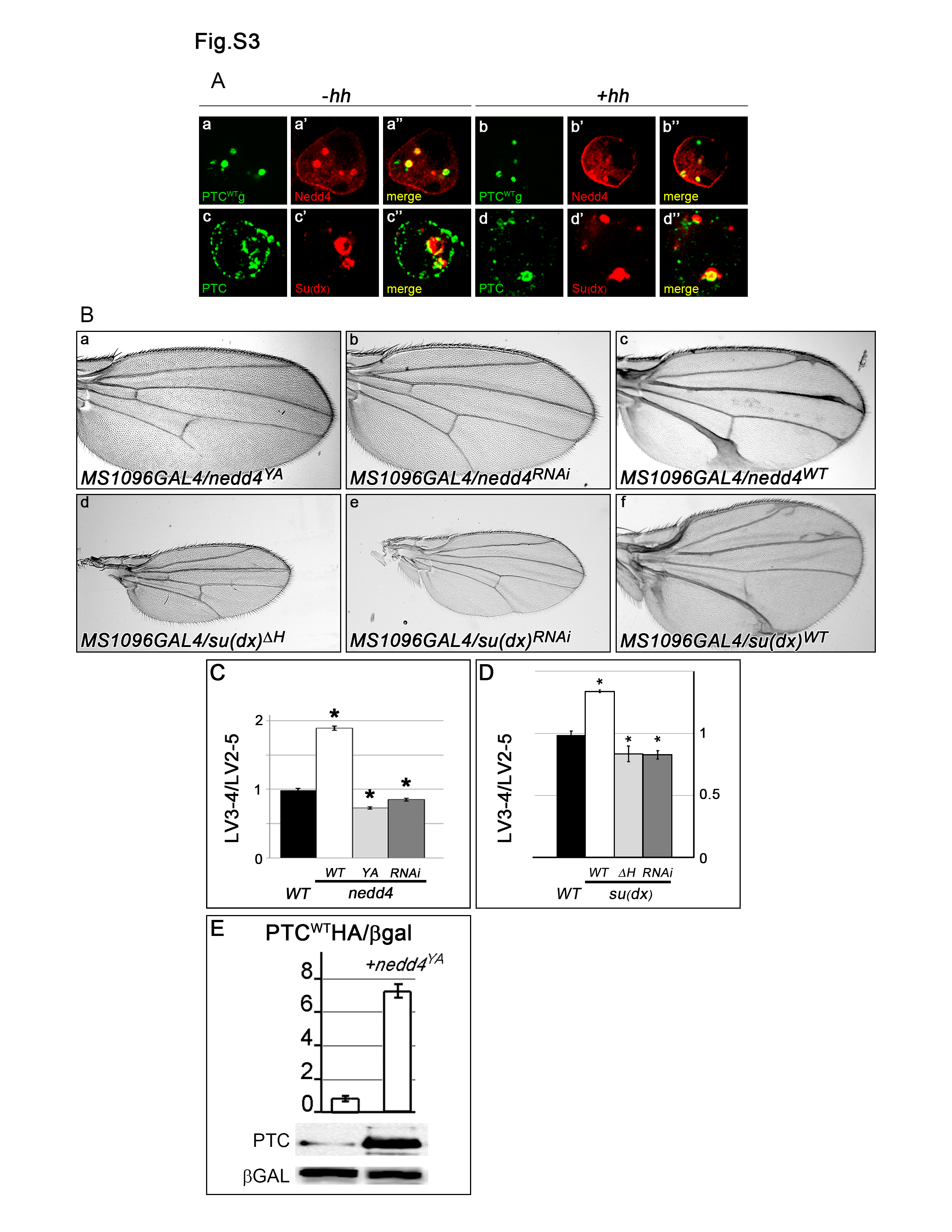

Supplement: Figure S3: NEDD4 and SU(DX) colocalize with PTC and their downregulation affects wing morphogenesis. [file rsob150112supp3.tif]

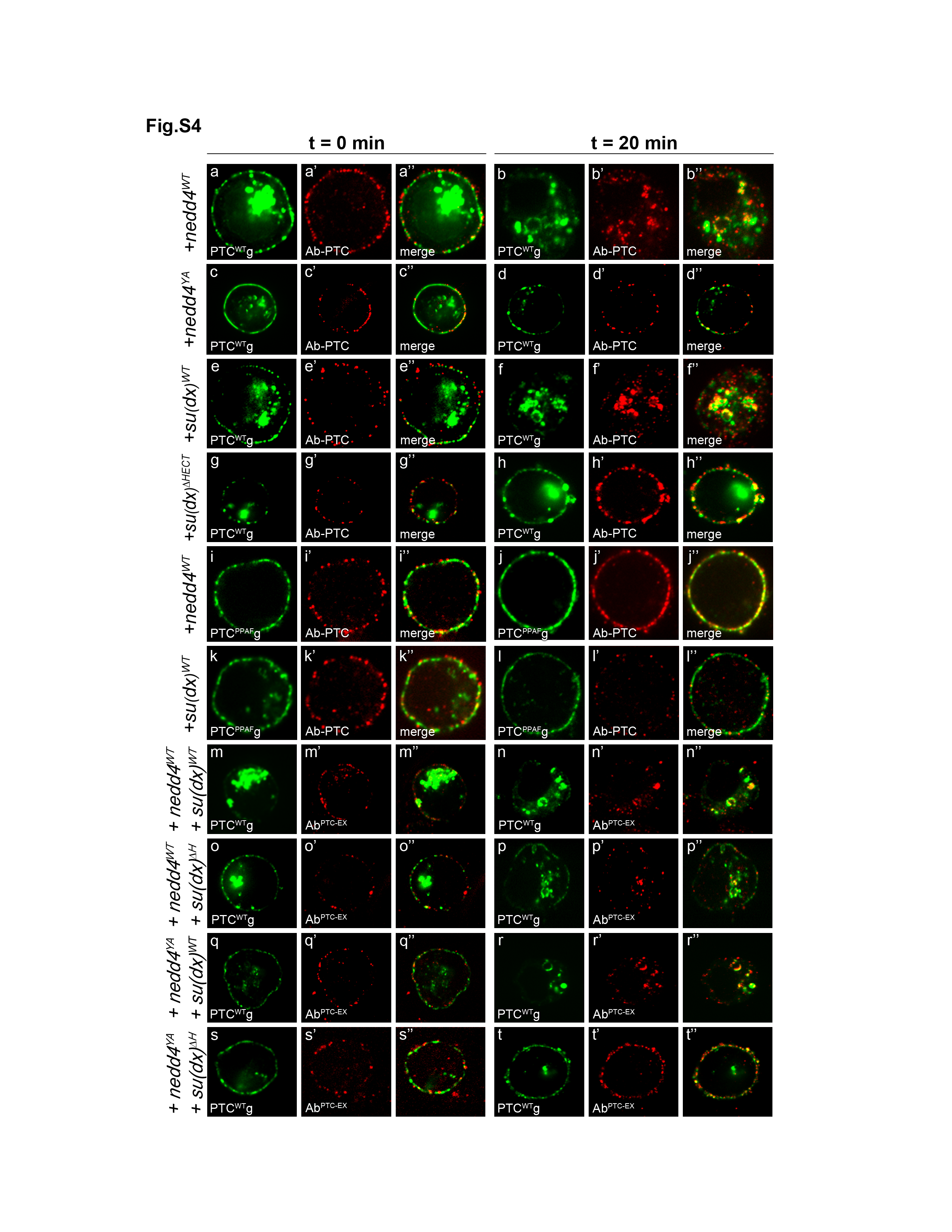

Supplement: (A) Images of S2 cells showing the colocalization of PTCWTg (green) and NEDD4 (immunolabeled in red) at the cell surface and in vesicles (a-b”) or of endogenous PTC (green) and SU(DX) (red) in vesicles (c-d”). Without HH (a-a”, c-c”) or with HH (b-b”, d-d”). (B-D) Wings from transgenic flies overexp [file rsob150112supp4.tif]
