# Supplementary material for: Control of the dynamics and homeostasis of the Drosophila Hedgehog receptor Patched by two C2-WW-HECT-E3 Ubiquitin ligases
Source: Open Biol. 2015 Oct 7;5(10):150112. doi: 10.1098/rsob.150112 (PMC4632511; doi:10.1098/rsob.150112)
Supplement: Table S1: Quantitication of the colocalization of PTCWTg with RAB5 or Lyso. [file rsob150112supp5.doc]

Table S1

| **RAB5** |  | **-** | | **+ HH** | |
| --- | --- | --- | --- | --- | --- |
|  |  | **PTC^WT^****g** | **random** | **PTC^WT^g** | **random** |
|  | **_** | 15.40  (+/- 1.10) | 3.44  (+/- 1.15) | 18.91  (+/- 0.98) | 4.15  (+/- 1.42) |
|  |  |  |  | t=9.28E-09 |  |
|  | **+ NEDD4^WT^** | 20.48  (+/- 1.00) | 2.95  (+/- 1.73) | 32.27  (+/- 2.29) | 3.56  (+/- 1.60) |
|  |  | t=1.57E-13* |  | t=5.89E-08** |  |
|  |  |  |  | t=2.43E-07 |  |
|  | **+ SU(DX)^WT^** | 18.50  (+/- 1.16) | 3.27  (+/- 1.61) | 20.91  (+/- 1.31) | 3.70  (+/- 1.82) |
|  |  | t=1.11E-09* |  | t=1.32E-04** |  |
|  |  |  |  | t=4.45E-04 |  |

| **Lyso** |  | **-** | | **+ HH** | |
| --- | --- | --- | --- | --- | --- |
|  |  | **PTC^WT^g** | **random** | **PTC^WT^g** | **random** |
|  | **-** | 3.13  (+/- 2.00) | 4.91  (+/- 0.76) | 24.60  (+/- 1.25) | 2.89  (+/-1.18) |
|  |  |  |  | t=1.26E-24 |  |
|  | **+ NEDD4^WT^** | 8.18  (+/- 0.87) | 3.40  (+/- 1.46) | 21.11  (+/- 1.09) | 2.15  (+/- 0.76) |
|  |  | t=3.69E-13* |  | t=1.61E-06** |  |
|  |  |  |  | t=3.97E-21 |  |
|  | **+ NEDD4^YA^** | 3.17  (+/- 1.16) | 3.30  (+/- 0.99) | 11.04  (+/- 0.72) | 3.50  (+/- 2.55) |
|  |  |  |  |  |  |
|  |  | t=9.38E-01 |  | t=5.15E-14** |  |
|  |  |  |  | t=1.47E-11 |  |
|  | **+SU(DX)^WT^** | 24.80  (+/- 0.74) | 4.14  (+/- 1.40) | 86.47  (+/- 4.08) | 2.79  (+/- 1.00) |
|  |  | t=3.02E-32* |  | t=1.28E-13** |  |
|  |  |  |  | t=1.27E-12 |  |
|  | **+SU(DX)^ΔH^** | 4.28  (+/- 0.87) | 3.83  (+/- 1.03) | 4.43  (+/- 1.01) | 4.29  (+/- 1.15) |
|  |  | t=1.95E-02 |  | t=3.93E-18** |  |
|  |  |  |  | t=6.95E-01 |  |
|  | **+ NEDD4^WT^**  **+ SU(DX)^WT^** | 21.75  (+/- 0.62) | 3.76  (+/- 1.02) |  | |
|  |  | t=2.93E-29* |  |  | |
|  | **+ NEDD4^WT^**  **+ SU(DX)^ΔH^** | 2.80  (+/- 0.92) | 3.40  (+/- 1.18) |  | |
|  |  | t=5.00E-01 |  |  | |
|  | **+ NEDD4^YA^**  **+ SU(DX)^WT^** | 22.03  (+/- 1.30) | 2.90  (+/- 1.35) |  | |
|  |  | t=1.49E-26* |  |  | |
|  | **+ NEDD4^YA^**  **+ SU(DX)^ΔH^** | 4.27  (+/- 0.86) | 3.63  (+/- 1.22) |  | |
|  |  | t=1.83E-02 |  |  | |
