# Supplementary material for: Control of the dynamics and homeostasis of the Drosophila Hedgehog receptor Patched by two C2-WW-HECT-E3 Ubiquitin ligases
Source: Open Biol. 2015 Oct 7;5(10):150112. doi: 10.1098/rsob.150112 (PMC4632511; doi:10.1098/rsob.150112)
Supplement: Table S1 legend.docx [file rsob150112supp6.docx]

**Table S1: Quantitication of the colocalization of PTC^WT^g with RAB5 or Lyso.**

To calculate the extent of co-localisation due to chance alone, PTC^WT^g images were rotated by ninety degrees respective to their matching vesicular marker images, and co-localisation with RAB5 and Lyso was re-analyzed (co-localization occurring by chance). Under these conditions, less than five percent of PTC randomly co-localized with any given marker. For each vesicular marker, 10 cells (25 images per stacks) randomly taken from three independent experiments were analyzed.

Boxes in gray indicate that the extent of colocalization of PTC^WT^g with the vesicular marker differed significantly from that expected by chance alone.

Blue boxes show PTC^WT^g colocalization with the vesicular marker, under the different conditions indicated, compared with wild-type conditions. Statistical significance (p<0.025) is indicated by * in the absence of HH and by ** in the presence of HH.

Red boxes compare the PTC^WT^g colocalization with the vesicular marker, under the conditions indicated, in the presence and absence of HH.
