# Supplementary material for: Control of the dynamics and homeostasis of the Drosophila Hedgehog receptor Patched by two C2-WW-HECT-E3 Ubiquitin ligases
Source: Open Biol. 2015 Oct 7;5(10):150112. doi: 10.1098/rsob.150112 (PMC4632511; doi:10.1098/rsob.150112)
Supplement: Sup mat and meth 8 sept 2015.docx [file rsob150112supp7.docx]

**Wings analysis:** ImageJ software was used to calculate the ratios widths of LV3-4 intervein region / LV2-5 intervein region. These ratios were compared to ratios for *MS1096* flies. Left wings of at least 10 males were analyzed for each genotype.

**Plasmid constructs:** *pAct5C-ptc^WT^-EGFP or -HA* and *pAct5C-ptc^PPAF^-EGFP or -HA, pUASt-GW-attB-ptc^WT^* and *pUASt-GW-attB-ptc^PPAF^* vectors were constructed by the Gateway (GW) recombination method (Life Technologies). PCR products obtained from the coding sequence (without the termination codon) of a *ptc^WT^* cDNA were inserted into pENTR/D-TOPO by directional TOPO Cloning. For *ptc^PPAF^*, an A to T replacement was introduced by site-directed mutagenesis at position 3626 of the *ptc* coding sequence (QuickChange Kit, Agilent technologies), WT and mutant *ptc* constructs were inserted into the following destinations vectors: *pAct5C-GW-EGFP*, *pAct5C-GW-HA* (gifts from T. Murphy) and *pUASt-GW-attB* constructed by insertion of the GW recombination cassette C3 at the EcoRI site of *pUASt-attB* (GI EF362409) [1]. *ptc^WT^-EGFP* has previously been shown to be functional [2]. *pUASt-nedd4^WT^* *pUASt-* was provided by S. Hayashi [3], *nedd4^YA^* and *pUASt-su(dx)^WT^* by M. Baron [4]. Deletion of the HECT domain was obtained by insertion of a PCR-amplified fragment from *su(dx)* cDNA, extending from codon 1 to 292. All PCR-amplified inserts were checked by sequencing. *pDA-hh* was provided by S. Cohen. *GST-su(dx)* constructs were given by M. Baron [4]. The C-terminal region of PTC (the 181 last aa) was expressed from pDEST15.

**Analysis of S2 cells and imaginal discs:** Schneider 2 (S2) cells transiently transfected [5] and dissected discs from third instar larvae were treated for indirect immunofluorescence, as described in [5]). Primary antibodies : mouse anti-PTC, 1/100 (Apa1 [6]), mouse anti-COL, 1/100 (Gift from M. Crozatier), mouse anti-SMO, 1/1000 (20C6, DSHB), rat anti-CI, 1/5 (2A1 [7]), rabbit anti-RAB5, 1/100 [8], rabbit anti-NEDD4, 1/2000 [3] and rabbit anti-SU(DX), 1/200 [4]. Secondary antibodies were from the Jackson Immuno Research Laboratory and were used at 1/200. Lysotracker Red DND-99 (Life Technologies) was incubated 2h at 25°C, and rinsed three times with PBS. Luciferase assays were carried out as described in [9] and repeated three times.

**Western blot analysis**: Transfected S2 cells were rinsed twice in PBS, lysed in RIPA buffer (50 mM Tris-HCl 1%, 0.15 M NaCl, 2 mM EDTA, 10% glycerol, 1% sodium deoxycholate, 0.1% SDS, 0.5% NP40, protease inhibitor (Complete Roche) and phosphatase inhibitor (Merck)) for 30 min at 4°C and centrifuged 10 min at 10000 rpm. Supernatants were diluted in loading buffer (Life Technologies) before electrophoresis and immunoblotting. Quantification was based on three independent transfections using Image J.

**GST pull-down assay**: GST-fusion protein production was induced by IPTG (2mM) 1h at 30°C to an exponential BL21 culture. Lysis using B-PER Reagent (ThermoScientific) with DNAse, anti-proteases and lysozyme was followed by sonication and centrifugation at 15000g. The supernatant was incubated for 1h at 4°C with GST beads and proteins produced *in vitro* using a TNT T7 system coupled with Fluorotech (Promega). After three washes (10 min at 4°C), the beads were mixed with 20µl of LDS sample buffer NuPAGE reducing agent before electrophoresis on NuPAGE 4-12% Bis-Tris gel (Life Technologies). Gels were scanned using a Typhoon imaging system, with 488 argon laser 532 nm excitation.

1 Bischof, J. *et al.* . 2007 *Proc Natl Acad Sci U S A*. **104**, 3312-3317.

2 Torroja, C. *et al.* . 2004 *Development*. **131**, 2395-2408.

3 Sakata, T. *et al.* 2004 *Curr Biol*. **14**, 2228-2236.

4 Wilkin, M. B. *et al*. 2004 *Curr Biol*. **14**, 2237-2244.

5 Claret, S. *et al.* 2007 *Curr Biol*. **17**, 1326-1333.

6 Capdevila, J., *et al.*1994 *Development*. **120**, 987-998.

7 Motzny, C. K., Holmgren, R. 1995 *Mech Dev*. **52**, 137-150.

8 Wucherpfennig, T., *et al.* 2003 *J Cell Biol*. **161**, 609-624.

9 Chen, C. H. *et al*. 1999. *Cell*. **98**, 305-316.
